# Development and effects of a webtoon education program on preventive self-management related to premature labor for women of childbearing age: a randomized controlled trial

**DOI:** 10.4069/kjwhn.2022.09.22

**Published:** 2022-09-30

**Authors:** Sun-Hee Kim

**Affiliations:** College of Nursing, Daegu Catholic University, Daegu, Korea

**Keywords:** Cartoons as topic, Health education, Knowledge, Premature obstetric labor, Self efficacy

## Abstract

**Purpose:**

The purpose of this study was to develop a webtoon education program on preventive self-management related to premature labor (PSM-PL) for women of childbearing age, to evaluate its effects, and to assess the usability of webtoon education for women of childbearing age.

**Methods:**

The study design was a stratified randomized trial with repeated measures. The participants were Korean women of childbearing age (between the ages of 19 and 49 years), with 49 participants each. The preventive health management self-efficacy related to premature labor (PHMSE-PL) scale, the preventive self-management knowledge related to premature labor (PSMK-PL) scale, and usability of webtoon education were assessed. The intervention group read six episodes of the PSM-PL webtoon within 2 days after clicking an online link. The control group did not receive anything but was given the webtoon after the last measurement. To test the effect of the repeatedly measured variables, a generalized estimating equation model was used.

**Results:**

The experimental group had statistically significantly greater increases in PHMSE-PL and PSMK-PL scores from baseline to immediately after and 2 weeks later than the control group. The average score for usability of webtoon education was high (4.52; standard deviation, 0.62) on a scale of 1–5.

**Conclusion:**

This webtoon education program on PSM-PL was a feasible and acceptable program that increased self-efficacy and knowledge of preventive health management of premature labor in women of childbearing age. Future studies that adopt a webtoon format can be beneficial for childbearing women with other risk factors.

## Introduction

On average, 5% to 18% of births worldwide are preterm [1]. Spontaneous premature labor is the most common cause of premature birth, accounting for 40% to 45% of cases, followed by premature labor after preterm premature rupture of membranes (25% to 30%) and various health problems in pregnant women and fetuses for which premature delivery is the best option rather than maintaining pregnancy [2]. In South Korea (hereafter, Korea), the proportion of patients receiving medical treatment for premature labor, relative to all births in the corresponding year, increased steadily from about 5.86% in 2016 to 6.61% in 2018 and 6.75% in 2020 [3-6]. In addition, medical expenses also increased from 37 billion Korean Won (KRW) (approximately 33 million US dollars) in 2016 to 46.5 billion KRW (roughly 42 million US dollars) in 2018 and 48.3 billion KRW (roughly 43 million US dollars) in 2020, despite the continuing decrease in the number of births [3-6].

Premature labor refers to the onset of uterine contractions before 37 weeks of pregnancy. A clinical diagnosis is made when cervical dilatation and effacement have progressed along with regular uterine contractions [7]. Premature labor can have a single cause, such as an infection in the amniotic fluid, but it can also result from multiple pathological processes involving multiple causes [8]. Premature labor does not always lead to premature birth, and full-term delivery is often possible with good prevention, early detection, and prompt management [7]. Since prepregnancy health status affects pregnancy outcomes, preventive management is necessary before pregnancy to improve pregnancy outcomes and reduce health care expenditures for mothers and newborns [9,10]. It is also necessary to identify risk factors for premature labor before pregnancy and manage them according to the degree of risk [7]. In other words, prepregnancy health care to prevent premature labor should not be limited to women who are already aware of and manage an underlying disease, but should also be received by women who are unaware of an underlying disease, have high-risk factors such as obesity and smoking, and have a history of premature labor and cervical surgery [9]. If a woman with high-risk factors becomes pregnant, she should receive regular, early prenatal care and proactive preventive treatment [9]. When receiving preconception care or after childbirth, it is an appropriate time to educate women who have high-risk factors for premature labor to control those factors [11]. In addition, even women at low risk for premature labor can prevent premature birth if they are aware of the symptoms of premature labor in advance and know how to respond appropriately [11]. Women of childbearing age should be able to learn about the risk factors of premature labor before conception in a self-directed way, to manage their health according to these risk factors and develop their preventive self-management capabilities.

Since the incidence of premature birth due to premature labor in developed countries is low, around 9% [1], most women expect and imagine only a normal pregnancy [12,13]. If women are not aware of the term “premature labor” in advance, they might first hear this phrase from a medical professional when they visit a hospital with abnormal symptoms of premature labor [12,13]. Therefore, the opportunity for an early and appropriate response may be missed at the time of symptom onset. A previous study found that women of childbearing age desired individually tailored prepregnancy health information, preferred sources of prepregnancy health information, and learned through the experiences of other women when planning pregnancy [14]. Education using webtoons, a type of digital comics that readers can access via computer or smartphones, can present various stories of women’s experience of premature labor and offer an alternative to personalized learning from others’ experiences.

The term “webtoon” was first used in Korea in 2000 [15] to refer to cartoons published online; these are also called digital comics or website cartoons [16,17]. Webtoons emerged as a comics genre unique to Korea and have now become a representative form and medium of the comics industry [15]. Webtoons are representative areas of the “snack culture” trend, and unlike printed comics, webtoons can be viewed conveniently in a short period of time, regardless of time and place, as long as the user has a device capable of accessing the internet [15]. Therefore, webtoons can be used as a medium for health education that can be easily spread in daily life [18]. In addition, webtoons were reported to have potential educational and cultural value due to their visual stimulation, narratives that can be practical and realistic. In other words, webtoons can provide a better understanding of factual information and give readers a greater understanding of the social and psychological aspects of the illness through the use of narrative, characterization, and images [18]. A prior study reported that using cartoons in the educational process provided learners with an educational environment that was enjoyable, interesting, and motivating to learn, and also facilitated comprehension, developed intelligence, ensured permanent learning, and facilitated evocatory [19]. In addition, webtoons were reported to have interactivity that draws upon their characteristics of openness, participation, and sharing [20,21]. Korean women of childbearing age are familiar with the use of internet devices and webtoons [15]. Furthermore, Korean women use the internet almost every day [22], and about 74% reportedly view webtoons at least once a week [23]. Therefore, Korean women of childbearing age may be open to webtoon learning, done on their own initiative at a time and place that is suitable for them.

To date, premature labor intervention studies have focused on early labor assessment and support at home [24], uterine monitoring for the detection of premature labor at home [25], support for women hospitalized for premature labor [26,27], and relaxation therapy for women hospitalized for premature labor [28-32]. These studies were all conducted among women hospitalized for premature labor, and most of them explored the use of relaxation therapy as a psychological intervention. No studies have yet targeted women at high risk of premature labor before pregnancy and before the onset of premature labor, and to our knowledge, no webtoon-based education on premature labor has been developed. Just as health care before pregnancy is recommended because prepregnancy health care affects pregnancy outcomes [9,10], preventive health education for women with high-risk factors for premature labor can affect pregnancy outcomes.

Therefore, this study developed a webtoon education program for preventive self-management related to premature labor (PSM-PL) for women of childbearing age and evaluated its effectiveness and usability to determine the future potential for wider applicability. The primary hypothesis of this study is as follows: the experimental group participating in the PSM-PL webtoon education program would have significantly higher levels of preventive health management self-efficacy related to premature labor (PHMSE-PL) than the control group immediately after education and at 2 weeks after baseline. The secondary hypothesis is that the experimental group participating in the PSM-PL webtoon education program would have significantly higher levels of preventive self-management knowledge related to premature labor (PSMK-PL) than the control group immediately after education and at 2 weeks after baseline.

## Methods

Ethics statement: This study was approved by the Institutional Review Board of Daegu Catholic University (CUIRB-2020-0031). Written consent was obtained from the participants, who indicated that they participated voluntarily. In addition, the same webtoon education program was provided through social networking services (SNS) to provide the control group with the same educational opportunity as the experimental group after the follow-up survey was completed.

### Study design

This study was a stratified randomized controlled design with parallel groups and repeated measures, with a 1:1 allocation ratio of the experimental and control group. The participants were stratified by age and the number of deliveries. The experimental and control groups were assigned using block randomization with a block size of four. This study was conducted in two stages; the first stage was the development of the webtoon education program, and the second stage was evaluation of its effectiveness and usability. The study adhered to the CONSORT (Consolidated Standards of Reporting Trials) guidelines (https://www.consort-statement.org/).

#### Development of the webtoon education program

First, the literature was reviewed on the prevention and self-management of premature labor for the purpose of improving self-efficacy and knowledge on preventive self-management of premature labor for women of childbearing age. For the literature search, MEDLINE, Cochrane Library, and CINAHL databases were used, for publications after 2010, and 62 studies were identified and reviewed. Text word and controlled language such as ‘premature labor,’ ‘prevention & control,’ ‘management,’ and ‘intervention’ were combined. The contents of education on risk factors of premature labor, preventive risk factor management, preventive daily life management, symptoms of premature labor, and early response methods when symptoms appear were extracted and preselected.

Next, an advisory group consisting of two nursing professors, four nurses who worked in the delivery room of a tertiary hospital, and one obstetrician finalized the contents by selecting important and relevant content and confirmed their validity. All seven experts were women, and the ages were 31 to 59 years old with 2 to 23 years of clinical experience in the field of women’s health. The experts advised adding support of family and acquaintances, which was thereby added to the educational content.

Six story themes were composed that addressed high-frequency triggers of premature labor. The educational contents to be included in the story of the main character for each topic were allocated. With the advice of one webtoon writer, the researcher directly wrote the title, background, and plot of the webtoon scenario. The title of the webtoon scenario was “*Early Detection of Premature Labor*,” and it was set in the background of a multi-person room in a high-risk maternity intensive care unit at a tertiary hospital in the modern era, in Korea, and in springtime; also, a sequential linear storytelling manner was chosen. Except for the selected educational contents, the personalities of all characters, the plot for each topic, and the overall plot composition were developed on a purely creative basis.

Based on the story, a webtoon draft was produced through a process of consultation with three webtoon writers. The content validity and comprehension of the webtoon were evaluated by five women of childbearing age, two nursing professors, and two obstetricians. The five women were aged 21–42 years old, four were married and had children, while one was unmarried and had never had children. The clinical experts were aged 34–56 years old, had 6–19 years of educational experience, and 5–20 years of clinical experience in women’s health field.

After modifying for facial expressions, actions, words, and the severity of the symptoms of premature labor according to the opinions of the webtoon draft evaluators, the webtoon education program was finalized. Table 1 shows the topics of the final webtoon education program (Supplementary Figure 1; https://posty.pe/sla9i0h.

#### Evaluation of the effectiveness and usability of the webtoon education program

##### 1) Participants

The inclusion criteria for participants were Korean women of childbearing age (between the ages of 19 and 49 years) who voluntarily agreed in writing to participate in the study. The exclusion criteria were a previous experience of premature birth due to premature labor or having learned about premature labor in nursing or medical college.

In this study, the primary endpoint was PHMSE-PL. There was no previous study that used the PHMSE-PL or self-efficacy in similar webtoon, comic, or cartoon programs. Therefore, the effect size of PHMSE-PL was set to the medium effect size to estimate the sample size in this study. Using G*Power version 3.1.0 [33] for the independent t-test to compare pre-post differences between the experimental group and the control group, and using a one-sided test, a significance level of .05, a power of .80, and an effect size of 0.50, the sample size was estimated as 51 women for each group. For repeated-measures analysis of variance, the total sample size was determined to be 34 when the power was .80, the significance level was .05, and the effect size was 0.25. Therefore, to ensure reliable statistical results after data collection, the target sample size was set at 102. Based on an assumed dropout rate of 10%, 114 women were considered adequate (57 in each group).

##### 2) Data collection/procedure

To recruit research participants, a data collection assistant posted a promotional message on an online community for pregnant women and women who had given birth (called ‘*Mom café*’) and through SNS to acquaintances. Applicants who wanted to participate voluntarily were guided to participate in the online survey (first round) by clicking the provided URL.

For data collection, an online survey administered through SNS was repeated three times at 2-week intervals from February 22 to March 24, 2021. For the first survey, participants were requested to read the research description online, click and sign the voluntary study participation agreement, check whether the inclusion criteria were met, and then click the URL to complete the first survey (time 0). The contents of the baseline survey were demographic and childbirth-related characteristics, PHMSE-PL, and PSMK-PL. One research assistant who did not participate in the experiment stratified the participants by age (20s, 30s, 40s) and the number of deliveries (0, 1, 2, 3, or more) from the baseline response data, and then applied random numbers to identify the participants in Excel (Microsoft, Redmond, WA, USA). Two weeks later, the experimental group received the webtoon URL through SNS and read the webtoon voluntarily within 2 days, after which they responded to the online survey (time 1). The second survey contained the PHMSE-PL scale, the PSMK-PL scale, and items on the usability of webtoon education. After 2 weeks, the third online survey was conducted (time 2), using the PHMSE-PL and PSMK-PL scales.

The control group was not offered anything but completed the same surveys at the same time as the experimental group, except for the usability of webtoon education. The control group received the webtoon URL after completing the third survey. Both the experimental and the control group received remuneration (worth 3 US dollars) each time they completed the questionnaire. Except for randomization, the first to third surveys and the provision of the webtoon education program were conducted by one researcher.

##### 3) Protocol adherence

**Figure 1.**
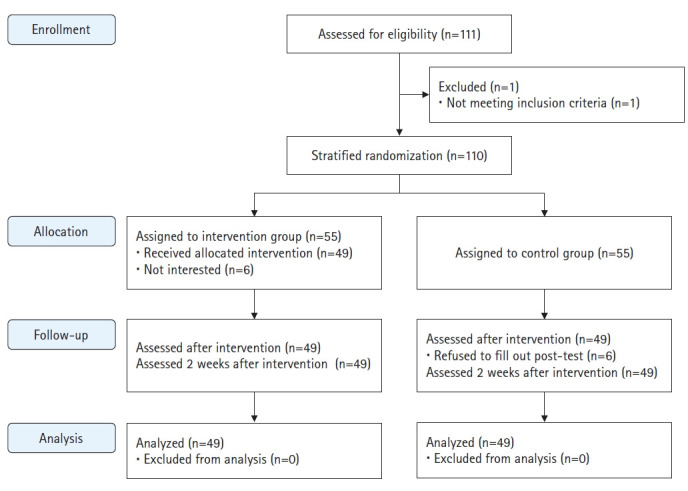
CONSORT diagram showing the flow of participants through the trial.

In total, 111 people participated in the first round of data collection. One of them experienced premature birth and was excluded. Therefore, 110 people were assigned to the experimental and the control groups (n=55 each). Two weeks after the first survey, 49 women (89.1%) in the experimental group responded to the second survey, and six did not complete all the webtoon educational materials or did not respond to the questionnaire by the deadline. In the control group, 49 women (89.1%) responded to the second survey, whereas six did not respond to the questionnaire. In the third survey, all 49 people in the experimental and the control group responded (Figure 1).

##### 4) Intervention

The webtoon education program on PSM-PL consisted of 30 to 52 squares per episode, with a total of six episodes, and could be viewed anywhere by clicking the link on an internet device. The webtoon was published on a blog created by the webtoon artist specifically for the publication of the “*Early Detection of Premature Labor*” webtoon, using TISTORY (https://www.tistory.com/), an open application programming interface provided by Kakao Corp. (Jeju, Korea). No other content was posted. In the preliminary survey, it took about 3 to 5 minutes to read each episode, and 18 to 30 minutes to read all six episodes. One author reached out to the participants in the experimental group individually through SNS, instructing them to use the URL to read all six webtoon episodes within 2 days.

##### 5) Measurements/instruments

###### (1) Preventive health management self-efficacy related to premature labor scale

The PHMSE-PL scale was developed by Kim and Lee [34], and its content validity and construct validity were confirmed. The scale contained 34 items across five subcategories. The subcategories were information-seeking about premature labor (seven items), preventive risk factor management (six items), preventive daily life management (six items), preventive high-risk health behavior management (three items), and early coping during symptom onset (twelve items). The responses to each item are on a Likert scale ranging from 1 (“I can’t do it at all”) to 5 (“I can do it very well”). The average ratings for each item are calculated. At the time of the tool’s development, Cronbach’s alpha for the overall PHMSE-PL was .96 [34], and .94 in this study.

###### (2) Preventive self-management knowledge related to premature labor scale

The PSMK-PL scale was developed by Kim and Lee [35], who evaluated the content validity using item response theory. The scale contained 24 items across three subcategories. The subcategories were risk factors for premature labor (ten items), preventive management (eight items), and symptoms and symptom management of premature labor (six items). Each item has a response of “correct” (1 point), “wrong” (0 points), and “do not know” (0 points). The total score is the sum of all item scores. At the time of the tool’s development, Cronbach’s alpha for the overall scale was .89 [35], and .88 in this study.

###### (3) Usability of webtoon education

For usability, to evaluate the user’s experience of webtoon education, the author developed and used items based on the honeycomb model [36]. This consisted of seven items: one item each for desirability, valuableness, accessibility, usefulness, convenience, merit for the future, and credibility of webtoon education. Each item has a response on a Likert scale ranging from 1 (“not at all likely”) to 5 (“extremely likely”). The average ratings for each item are calculated. Cronbach’s alpha of the overall tool was .97 in this study.

##### 6) Data analysis

The collected data were analyzed using IBM SPSS ver. 25 (IBM Corp., Armonk, NY, USA). The frequency, percentage, mean, and standard deviation (SD) were calculated to identify the general characteristics of the participants, and the normality of the distribution of data for the variables was evaluated with the Shapiro-Wilk test. The general characteristics of the experimental and control groups showed a non-normal distribution; therefore, the chi-square test and the Mann-Whitney U-test were used to verify the homogeneity. The major effect measurement variables (PHMSE-PL, PSMK-PL, usability of webtoon education) were presented as the mean and SD. The baseline scores of the major effect measurement variables were normally distributed, the t-test was used for the baseline homogeneity test, and all scores of the second and third-time points of the experimental and control groups were not normally distributed. Therefore, to test the effects of the repeatedly measured variables, a generalized estimating equation model was used. The results of the model were presented as the model coefficient (β), standard error, and 95% confidence interval. The reliability of all measurement tools was analyzed using Cronbach’s α. All hypothesis tests involved a one-sided significance level of α=.025, and the baseline homogeneity test between two groups involved a two-sided significance level of α=.05.

## Results

### Development of the webtoon education program

The themes and contents of the webtoon education program are shown in Table 1. The theme of episode 1 was that prolonged excessive activities in the early third trimester are a risk factor for premature labor. The theme of episode 2 was that genital infection and excessive activity at the end of the second trimester is a risk factors for premature labor. The theme of episode 3 was that a twin pregnancy at the beginning of the third trimester with a history of premature labor is a risk factor for premature labor. The theme of episode 4 focused on excessive amniotic fluid and amniotic membrane rupture due to gestational diabetes, as risk factors for premature labor. The theme of episode 5 was on intrauterine infection as a cause of premature birth. Finally, the theme of episode 6 focused on progesterone medication in the treatment of cervical incompetence to prevent premature labor and being sensitive to the symptoms of premature labor.

### Evaluation of the effectiveness and usability of the webtoon education program

#### Comparison of demographic and childbirth characteristics and outcome variables at baseline

The average age of the participants was 30.60 years (SD, 8.31 years); 56.1% were college graduates, 57.3% of the participants had no job, and 54.1% of respondents were single. Most participants (92.9%) were not currently pregnant, and the average number of pregnancies of respondents was 0.84 (SD, 1.13). Most of the women who had been pregnant (83.3%) had no pregnancy complications. The average number of deliveries was 0.65 (SD, 0.89) times, their average number of children was 0.66 (SD, 0.90), and 40.8% had experienced pregnancy and childbirth education. More than half (58.3%) reported having viewed webtoons before.

There were no statistically significant differences between the experimental and control groups in any general characteristics (Table 2). In addition, there were no statistically significant differences in the PHMSE-PL (t=–0.33, *p*=.745) and the PHMK-PL (t=–0.36, *p*=.717) between the experimental and control groups at baseline (Table 3).

#### Preventive health management self-efficacy related to premature labor

Table 3 shows the PHMSE-PL scores before the webtoon education program (time 0), immediately after (time 1), and 2 weeks after (time 2) the webtoon education program on PSM-PL. The intervention group showed significantly greater improvements in PHMSE-PL at both time 1 (χ^2^=8.29, *p*=.004) and time 2 (χ^2^=6.27, *p*=.012) with respect to time 0 than the control group (Table 4).

#### Preventive self-management knowledge related to premature labor

Table 3 shows PSMK-PL scores at time 0, time 1, and time 2 of the webtoon education program on PSM-PL. The intervention group showed significantly greater improvements in PSMK-PL at both time 1 (χ^2^=20.09, *p*<.001) and time 2 (χ^2^=14.42, *p*<.001) with respect to time 0, when compared to the control group (Table 4).

#### Usability of webtoon education for preventive self-management related to premature labor

The average score for usability of webtoon education for PSM-PL for all questions was high at 4.52 (SD, 0.62), and usefulness and convenience had the highest scores (4.57 [SD, 0.61] for both), while desirability had the lowest score (4.43 [SD, 0.82]) (Table 5).

## Discussion

This webtoon education program consists of a total of six episodes and 200 number of squares, telling the story of five pregnant women with premature labor. The risk factors for premature labor in these women were selected as the most common cases based on the literature and expert opinions, and the risk factors were different for each woman. This webtoon education program included risk factors of premature labor, preventive risk factor management, preventive daily life management, symptoms of premature labor, early response methods when symptoms appear, and support from family and self-help groups. Because webtoons can be accessed easily through smartphones, computers, and laptops through the internet, they have the advantage of being conveniently viewed in a short time [17]. Thus, webtoons are a valuable medium for health education in places where the internet is easy to access, such as in Korea [18]. Humans most naturally organize their experiences and knowledge through the narrative form [37]. Narrative learning helps students to obtain learning results through the process of exploring and solving problems at the individual or social level in various stories that already exist in the real-life context [37]. Webtoons not only deliver story-based learning content, but also foster self-awareness, reassurance, empathy, and companionship due to the cartoon elements of characters and images [18]. Webtoons are also a valuable health information medium [18] because they facilitate improvements in academic interest, immersion, learning [20,38], and metacognition [20]. Therefore, as a narrative medium based on realistic stories, webtoons help readers naturally organize the delivered knowledge [37]. It is also possible to create metacognitive sensitivity that can be applied in close connection with the real-world context of one’s life through the narrative learning method [37]. The present study developed and administered a webtoon-based intervention because of its advantages in terms of both learning effects and the delivery method. Since the main characters were ordinary women who presented their individual experiences of premature labor as a story, indirect experiential learning was naturally induced while participants read the story, instead of a one-sided method of passively receiving information from the educator. Many educational materials have been developed using cartoons, but these have not generally presented realistic stories; instead, they have generally used formats such as an educator character presenting one-sided explanations [39], story-based comic books [38,39], cartoon media characters presenting information [40], and video formats [41]. Unlike the passive nature of these formats, webtoons allow posting opinions and “likes” which encourage bidirectional engagement, while presenting stories that readers can relate to. A recent study used a webtoon to provide education on COVID-19 infection prevention and reported that webtoon education had an educational effect on knowledge development and positive attitude formation through emotions and inducing preventive behavior [42]. Meanwhile, another study reported that women of childbearing age wished for individually tailored prepregnancy health information, preferred sources of prepregnancy health information, and preferred learning through the experiences of other women when planning pregnancy [14]. In medical education, both instructors and students preferred cartoons because they believed that cartoons create a positive learning environment, support students’ thinking, and help promote verbal communication between instructors and students [43]. Therefore, since the webtoon education program presented in this study was personalized in time and place and involved learning through case experiences, it aligns with what is expected as a preferred source of health information.

In this study, the experimental group had statistically significantly higher PHMSE-PL than the control group immediately after participating in the education program and after 2 weeks. In previous studies, prenatal education using comics was found to be partially effective in improving the environmental health behaviors of pregnant women [39]. Narrative-based educational information from comic books was more effective than traditional information-based education on pregnant women’s self-efficacy in reducing climate change-related health risks [44]. Because the participants of this study participated in story-based narrative learning using webtoons, we can posit that their learning was internalized by considering high-risk factors of premature labor in the context of an individual’s life and how to seek solutions [37]. The visual images of the webtoon and the personalities of the main characters also likely contributed to enhancing the learning effect [20,38].

Furthermore, in this study, the experimental group had a statistically significantly higher PSMK-PL than the control group immediately after participation in the educational program and after 2 weeks. In previous studies, narrative-based educational information from comic books had an effect on pregnant women’s knowledge of health risks related to climate change [44] and on knowledge about the prevention and management of COVID-19 [42]. Cartoons were found to be valuable as a health information medium [18] and had an effect on students’ academic achievement in social studies [21]. It has also been argued that cartoon mnemonics are time-efficient for medical students who must memorize many facts [45]. Therefore, webtoons are thought to improve knowledge by increasing learning interest and learning immersion, making information easier to remember [20,38].

A meaningful aspect of this study is that it evaluated the usability of the webtoon health education related to premature labor based on stories, since this is one of the first studies to use a webtoon-based study to provide health information to women of childbearing age. The average score for usability of webtoon education in this study was high at 4.52 points, with generally high scores ranging from 4.43 to 4.57 across the subcategories. Particularly high scores were found for usefulness, valuableness, and merit for the future of webtoon education, reflecting the recognition that this webtoon presented effective educational material. Since the material was published as a webtoon and only one participant did not view it within the given time, it is presumed that it was convenient and accessible. In this webtoon, however, only four colors (including black and white) were used, and digital effects were not used [46], which may explain why desirability had the lowest score.

In this study, webtoon scenarios describing five cases of premature labor were composed, and their effects were confirmed. In the future, it will be necessary to develop various cases, create a program that can induce behavioral changes as well as improve self-efficacy and knowledge, and conduct evaluation studies. Another point worth noting is that the characters in the webtoons were portrayed realistically to enhance empathy and immersion. However, there are limitations in interpreting the effects of the webtoons in terms of empathy and immersion, because it was not confirmed which specific characteristics of the webtoons affected participants’ learning modalities. The webtoons developed in this study could be individually tailored in terms of time and place, but the educational content was not specifically personalized.

Some possible options for creating a truly personalized online webtoon education program and deepening virtual experiential learning could include developing an interactive webtoon story in which the user directly selects relevant options of risk factors for premature labor and creates the story, using movement technology for characters, or applying the technology of a three-dimensional spatial experience in which the user enters the story [16].

Blinding was not performed due to the characteristics of the webtoon education program in this study, and it was not possible to confirm long-term effects such as preventive health management practices for premature labor during actual pregnancy and indicators related to the direct occurrence of premature labor. Future research to confirm long-term, practical effects are suggested. The time of retest in this study was as short as 2 weeks after the intervention. In future studies, it is suggested to measure the effect by extending the time of the retest. Since there was no pre-existing measurement tool suitable for evaluating the effectiveness of this program, the measurement tools used in this study were not widely utilized. The interpretation of the research results is limited because these tools have not been applied to various groups or sufficiently tested for validity and reliability. It is meaningful that this study evaluated the effectiveness of the webtoon education program on PSM-PL for the first time in nursing. Unlike animation-based educational materials containing stories, which require substantial investments of time and money, webtoon educational materials are relatively time- and cost-effective. No study has yet compared the educational effectiveness of animations and webtoons, so it is expected that a comparative study on time, cost, and educational effects will be conducted in the future.

In conclusion, this study confirmed that a webtoon education program could potentially improve self-efficacy and knowledge of health management related to premature labor in women of childbearing age. As this webtoon education program was also feasible and evaluated as useful, clinicians can use it at various health education sites in the future to enhance the self-management capacity of women of childbearing age to prevent premature labor.

## Figures and Tables

**Table 1.** Main themes and contents of the webtoon stories

Episode	Themes	Contents	No. of squares
1	Prolonged excessive activities in the early third trimester are a risk factor for premature labor	‧ Introduction to a maternal-fetal intensive care unit	52
		‧ Risk factors of premature labor: prolonged excessive activities in the early third trimester	
		‧ Medical diagnostic tests: non-stress test, ultrasound to check the cervical length, vaginal discharge analysis	
		‧ Hospitalization procedure in the maternal-fetal intensive care unit and inpatient life	
		‧ Medical treatment: corticosteroid treatment to accelerate fetal lung maturation and treatment with medications to relieve uterine contractions	
		‧ Prevention and early management	
		- Distinguishing between false and true labor pain	
		- Reduce body fatigue during pregnancy, adjust daily activities or tasks, and follow a regular daily life so as not to overwhelm the body	
		- If you suspect premature labor, contact or visit the hospital immediately	
		- If premature labor is suspected, the degree of shortening of the cervix is evaluated, and a labor test and a vaginal discharge test are immediately conducted at the hospital	
		‧ Family support promotes psychological stability	
2	Genital infections and excessive activity at the end of the second trimester are risk factors for premature labor	‧ Risk factors of premature labor: genital infections or urinary tract infections that have not been completely treated, physically vigorous activity, and intensive work	30
		‧ Medical diagnostic tests: non-stress test, ultrasound, vaginal discharge analysis	
		‧ Medical treatment: antibiotics, corticosteroids to accelerate fetal lung maturation, and tocolytic drugs	
		‧ Prevention and early management	
		- If you have a genital infection that causes premature labor, visit a hospital frequently to manage it and treat it completely	
		- Distinguish between false and real labor pain	
		- Take a break immediately when you first notice the symptoms of premature labor	
		- If you suspect premature labor, contact or visit the hospital immediately	
		‧ Support from self-help groups promotes psychological stability	
3	A twin pregnancy at the beginning of the third trimester with a history of premature labor is a risk factor for premature labor	‧ Risk factors of premature labor: multiple pregnancies, short birth intervals	28
		‧ Medical diagnostic test: ultrasound: to check the degree of shortening of the cervix	
		‧ Medical treatment	
		- Pregnant women with a short cervix are administered progesterone starting in the second trimester	
		- Tocolytic drugs and their side effects	
		‧ Prevention and early management	
		- If you are pregnant with multiple gestations, visit the hospital frequently for management	
		- Sensitive observation and protection of the body during pregnancy	
		- Self-check for uterine contractions during pregnancy	
		- If you suspect premature labor, contact or visit the hospital immediately	
		‧ Support from self-help groups promotes psychological stability	
4	Excessive amniotic fluid and amniotic membrane rupture due to gestational diabetes are risk factors for premature labor	‧ Risk factors of premature labor: excessive amniotic fluid caused by gestational diabetes causes excessive enlargement of the uterus, amniotic membrane rupture, and psychological stress	28
		‧ Medical treatment: antibiotics, corticosteroids for accelerating fetal lung maturation, tocolytic drugs, and minimal daily activities at a hospital	
		‧ Prevention and early management	
		- If you have a disease that causes premature labor (excessive amniotic fluid, premature rupture of the membrane), visit a hospital frequently to manage it	
		- If there is a risk factor for premature labor, avoid severe exercise as much as possible	
		- Control psychological stress to prevent premature labor	
		- If you suspect premature labor, or leakage of the amniotic fluid, contact or visit the hospital immediately	
		‧ Support from family and self-help groups promotes psychological stability	
5	Intrauterine infection is a cause of premature birth	‧ Risk factors of premature birth: intrauterine infection	33
		‧ Medical treatment: premature delivery by cesarean section	
		‧ Support from health professionals, family, and self-help groups promotes psychological stability	
6	Progesterone medication in the treatment of cervical incompetence prevent premature labor	‧ Risk factors of premature labor: past experience of premature birth, short cervix length, and physically excessive activity	29
	Be sensitive to the symptoms of premature labor	‧ Medical diagnostic test: ultrasound	
		‧ Preventive medical treatment: progesterone hormonal treatment	
		‧ Prevention and early management	
		- Sensitive observation and protection of the body during pregnancy	
		- Follow a regular daily routine so that the body is not overwhelmed	
		- Distinguish between false and true labor pain	
		- If premature labor is suspected, carefully observe the changes in labor pain	
		- If you suspect premature labor, contact or visit the hospital immediately	

**Table 2.** Homogeneity of general characteristics between two groups (N=98)

Variable	Categories	n (%) or mean±SD			Test statistics	*p*
		Total (n=98)	Exp (n=49)	Cont (n=49)		
Age (years)^†^		30.60±8.31	30.31±8.57	30.90±8.12	1247.50	.738
Education^‡^	High school or below	36 (36.7)	21 (42.9)	15 (30.6)	2.07	.352
	Bachelor	55 (56.1)	24 (49.0)	31 (63.3)		
	Master or more	7 (7.2)	4 (8.2)	3 (6.1)		
Occupation^‡,§^	None	55 (57.3)	29 (59.2)	26 (55.3)	7.24	.059
	Permanent position	21 (21.9)	11 (22.4)	10 (21.3)		
	Temporary position	11 (11.4)	2 (4.1)	9 (19.1)		
	Others (self-employment)	9 (9.4)	7 (14.3)	2 (4.3)		
Marital status	Single	53 (54.1)	26 (53.1)	27 (55.1)	0.04	.839
	Married	45 (45.9)	23 (46.9)	22 (44.9)		
Currently pregnant^‡^	Yes	7 (7.1)	5 (10.2)	2 (4.1)	-	.436
	No	91 (92.9)	44(89.8)	47 (95.9)		
Number of pregnancies^†^		0.84±1.13	0.86±1.15	0.82±0.11	1,193.00	.953
Experience of pregnancy complications among those who had been pregnant^‡^	Yes	7 (16.7)	3 (15.8)	4 (17.4)	-	>.999
	No	35 (83.3)	16 (84.2)	19 (82.6)		
Number of deliveries^†^		0.65±0.89	0.65±0.90	0.65±0.88	1,210.00	.939
Number of children^†^		0.66±0.90	0.65±0.90	0.67±0.90	1,219.00	.881
Educated experience in pregnancy and childbirth	Yes	40 (40.8)	20 (40.8)	20 (40.8)	<0.001	>.999
	No	58 (59.2)	29 (59.2)	29 (59.2)		
Experience in reading webtoons	Yes	57 (58.2)	29 (59.2)	28 (57.1)	0.04	.838
	No	41 (41.8)	20 (40.8)	21 (42.9)		

Cont: Control group; Exp: Experimental group.

† Mann-Whitney U-test,

‡ Fisher exact test.

§ Data were analyzed with the number of controls set to 47 due to the two missing values.

**Table 3.** Mean and standard deviation values of outcome variables between both groups across study time points and the baseline comparisons (N=98)

Variable	Time	Experimental group (n=49)		Control group (n=49)		Comparison of groups at T0, t (*p*)^†^
		Mean	SD	Mean	SD	
PHMSE-PL	Time 0	3.92	0.57	3.95	0.50	–0.33 (.745)
	Time 1	4.26	0.58	4.02	0.59	
	Time 2	4.37	0.45	4.15	0.60	
PSMK-PL	Time 0	15.53	4.77	15.86	4.11	–0.36 (.717)
	Time 1	21.00	2.32	17.41	4.02	
	Time 2	21.49	2.75	18.39	4.40	

PHMSE-PL: Preventive health management self-efficacy related to premature labor; PSMK-PL: preventive self-management knowledge related to premature labor; Time 0 (T0): baseline test; Time 1: after education; Time 2: 2 weeks after education.

† Independent t-test between experimental and control group.

**Table 4.** Generalized estimating equation analysis regarding the effects of webtoon education on preventive self-management related to premature labor (N=98)

Variable	Categories	Beta	SE	95% Confidence interval		Wald χ^2^	One-sided
				Lower	Upper		*p*
PHMSE-PL	Group^†^						
	Exp	–0.01	0.027	–0.06	0.04	0.11	.371
	Time^‡^						
	Time 1	0.02	.014	–0.01	0.04	1.4	.119
	Time 2	0.05	.017	0.02	0.08	8. 88	.003
	Group×time^§^						
	Group (Exp)×time 1	0.07	.024	0.02	0.11	8.29	.002
	Group (Exp)×time 2	0.06	.024	0.01	0.11	6.27	.006
PSMK-PL	Group^†^						
	Exp	–0.02	.06	–0.13	0.09	0.13	.357
	Time^‡^						
	Time 1	0.09	.03	0.04	0.15	12.32	<.001
	Time 2	0.15	.03	0.1	0.2	31.97	<.001
	Group×time^§^						
	Group (Exp)×time 1	0.21	.05	0.12	0.3	20.09	<.001
	Group (Exp)×time 2	0.18	.05	0.09	0.27	14.42	<.001

Exp: Experimental group; PHMSE-PL: preventive health management self-efficacy related to premature labor; PSMK-PL: preventive self-management knowledge related to premature labor; time 1: after education; time 2: 2 weeks after education.

† Reference was control group;

‡ reference was baseline score;

§ references were Exp group×time 0 and control group×time 0, 1, and 2.

**Table 5.** Usability of webtoon education for preventive self-management related to preterm labor (N=49)

Item	Mean	SD
Desirability	4.43	0.82
Valuableness	4.51	0.79
Accessibility	4.55	0.58
Usefulness	4.57	0.61
Convenience	4.57	0.61
Merit for the future	4.51	0.62
Credibility	4.49	0.65
Total items	4.52	0.62

## Supplementary materials

Further details on supplementary materials are presented online (available at https://doi.org/10.4069/kjwhn.2022.09.22).

Supplementary Figure 1. Part of webtoon episode 1. Please see the linked webtoon platform (https://driedseaweed53.postype.com/series/743232/조기진단-조기진통).
