# Supplementary material for: Development and effects of a webtoon education program on preventive self-management related to premature labor for women of childbearing age: a randomized controlled trial
Source: Korean J Women Health Nurs. 2022 Sep 30;28(3):250–63. doi: 10.4069/kjwhn.2022.09.22 (PMC9619162; doi:10.4069/kjwhn.2022.09.22)
Supplement: Supplementary Figure 1. — Part of webtoon episode 1. Please see the linked webtoon platform (https://driedseaweed53.postype.com/series/743232/조기진단-조기진통). [file kjwhn-2022-09-22-suppl.pdf]

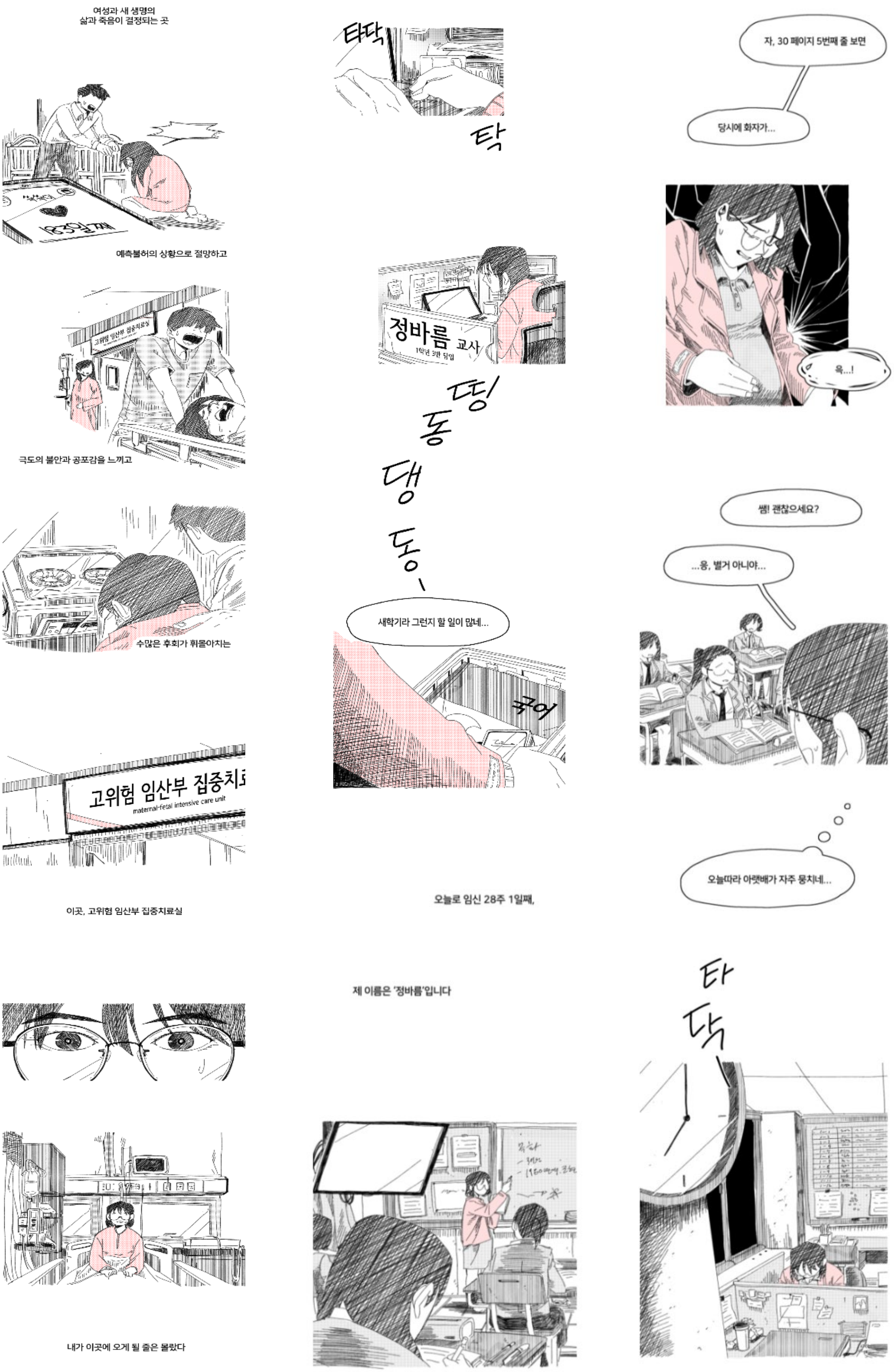

Supplementary Figure 1. Part of webtoon episode 1. Please see the linked webtoon platform (https://posty.pe/sla9i0h).
